# Molecular changes in the medial prefrontal cortex and nucleus accumbens are associated with blocking the behavioral sensitization to cocaine

**DOI:** 10.1038/srep16172

**Published:** 2015-11-05

**Authors:** Yi Zhang, Xiongzhao Zhu, Can Huang, Xiuwu Zhang

**Affiliations:** 1 Medical Psychological institute, Second Xiangya Hospital, Central South University, Changsha, Hunan 410011, P.R. China; 2 Department of Radiation Oncology, University of Maryland School of Medicine, Baltimore, MD 21221, USA; 3 Department of Cardio-thoracic Surgery, Second Xiangya Hospital, Central South University, Changsha, Hunan 410011, P.R. China

## Abstract

Previous studies have demonstrated that cocaine-induced behavioral sensitization is associated with persistent functional and structural alterations in the medial prefrontal cortex (mPFC) and nucleus accumbens (NAc); however, the molecular mechanisms underlying these changes have not been elucidated. In this study, the behavioral sensitization to cocaine was established in Sprague Dawley rats and was measured by locomotion and behavioral rating. The brain tissue homogenization was used for measuring the level of brain-derived neurotrophic factor (BDNF), the expression and activity of integrin-linked kinase (ILK), level of protein kinase B (Akt) phosphorylation at serine 473 and threonine 308, and the expression of p75^NTR^, TrkA, and TrkB protein. The Results showed that cocaine sensitization was associated with increased BDNF, ILK activity, phospho-Akt Ser^473^, p75^NTR^, and TrkB protein levels in the mPFC and NAc core. The combination of pergolide and ondansetron normalized not only behavioral sensitization, but also the increases in these molecular markers. Dual immunofluoresence staining showed that ILK expression is co-distributed with p75^NTR^ and TrkA expression in both the mPFC and NAc core. Results suggested that the BDNF-TrkA/p75^NTR^-ILK-Akt signaling pathway may be active in cocaine sensitization and associated neural plasticity in the mPFC and NAc core.

Although numerous preclinical and clinical studies have been conducted to identify treatment regimens that promote long-term abstinence in chronic cocaine abusers, few treatments have shown consistent efficacy^1^. This is because the molecular mechanisms driving the alterations in neurological plasticity commonly seen in cocaine abusers, related to their vulnerability to episodes of craving, have not been elucidated^2^. Thus, elucidation of the molecular basis of this vulnerability is expected to help identify effective treatment targets. Previous studies have demonstrated that brain-derived neurotrophic factor (BDNF) is a key protein involved in synaptic plasticity within the central nervous system^3^,^4^. BDNF is also implicated in long-term behavioral sensitization to psychostimulants^5^,^6^,^7^. For example, withdrawal from cocaine self-administration significantly increases the levels of BDNF in the ventral tegmental area (VTA), nucleus accumbens (NAc), and amygdala^8^. In addition, direct infusion of BDNF into the VTA or dorsomedial prefrontal cortex of rodents induced long-lasting enhancement of cocaine seeking^9^ or cocaine self-administration-induced elevation of p-synapsin in nucleus accumbens^10^. BDNF has been demonstrated to exert its effects through activation of various signal transduction pathways, including those involving phosphatidylinositol-3-kinase (PI3 K), mitogen-activated kinase (MAPK), and phospholipase C-γ^11^,^12^.

Currently identified neurotrophin receptors include the tyrosine kinase (Trk) receptors, such as TrkA, TrkB, TrkC, and the common neurotrophin receptor p75^NTR^^13^. Different neurotrophins have been shown to interact differently with these receptors. For example, all proneurotrophins and matured neurotrophins bind to and activate p75^NTR^. In contrast, no proneurotrophin binds to Trk receptors. The mature neurotrophins exhibit more specific interaction with the 3 Trk receptors, such as the main interaction of NGF with TrkA, the interaction of BDNF and NT4 with TrkB, and the interaction of NT3 with TrkC^14^. However, BDNF has been revealed to bind all neurotrophin receptors^13^. It has been revealed that p75^NTR^ can be found in a complex with Trk receptors to conduct neurotrophin signaling for neural survival^15^. For example, a study in PC12 cells showed that co-expression of p75^NTR^ with TrkA can enhance NGF-induced neurite outgrowth and survival^16^. Another study in oligodendrocytes showed that treatment with BDNF can stimulate p75^NTR^ and TrkA, but not TrkB receptor expression^17^.

The available evidence suggests that alterations in PI3 K/protein kinase B (Akt) signaling may contribute to the expression and maintenance of behavioral sensitization^18^,^19^. PI3K-mediated full activation of Akt requires phosphorylation at both the Thr^308^ and Ser^473^ residues. Besides PI3K, integrin-linked kinase (ILK), an upstream kinase of Akt, is also responsible for PI3K-dependent Akt Ser^473^ phosphorylation^20^. ILK has been demonstrated to be located in neuronal cell bodies and dendrites in various brain regions^21^,^22^. Furthermore, ILK may play key roles in divergent processes in the central nervous system, such as neurite outgrowth, survival of neurons, and myelination by oligodendrocytes^23^,^24^,^25^,^26^.

Previous studies have demonstrated that the 5-HT_3_ antagonist ondansetron or the D_1_/D_2_ agonist pergolide, when given after cocaine administration, can reverse previously-established cocaine sensitization^27^,^28^ with a reversal in the expression of selected molecular markers, such as NMDA (N-methyl-D-aspartic acid) and AMPA (alpha-amino-3-hydroxy-5-methyl-4-isoxazolepropionic acid) receptors^28^. In the present study, we investigated whether the levels of BDNF, TrkA, TrkB, p75^NTR^, Akt, and ILK proteins also exhibit characteristic changes following the establishment of cocaine sensitization and whether their expressions could be blocked.

## Materials and Methods

### Animals and Drugs

Male Sprague-Dawley rats (SLAC LABORATORY ANIMAL Inc., Shanghai, China), initially weighing 150–200 g, were housed in pairs in the animal facility of Central South University on a 12-h light/dark cycle for 1 week prior to the experiments. Rats were then housed individually after initial vehicle or cocaine injection. All experiments were conducted in accordance with the National Institutes of Health Guide for the Care and Use of Laboratory Animals and Chinese legislation on the use and care of laboratory animals. All efforts were made to minimize animal suffering, to reduce the number of animals used, and to utilize alternatives to *in vivo* techniques. All experimental protocol were approved by the Institutional Committee of Central South University

Cocaine HCl (Qinghai Pharmaceutical Factory, China), ondansetron hydrochloride dihydrate (GlaxoSmithkline, Research Triangle Park, NC), and pergolide (Sigma, St. Louis, MO) were prepared as described^28^.

### Experimental Groups and Behavioral Measurements

**Figure 1:**
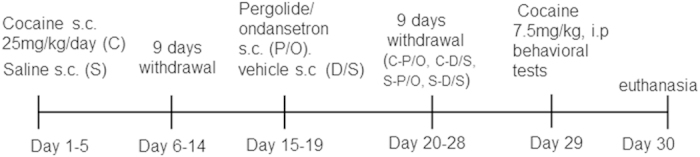
A schematic timeline on the behavioral experiments and experimental design.

Animals were treated as previously described^28^. Briefly, rats received 25 mg/kg/day cocaine (s.c.) for 5 consecutive days, followed by withdrawal for 9 days to establish long-term cocaine sensitization, or rats received saline injections plus 9 days of withdrawal. Starting on day 10 of withdrawal, half of the rats that received cocaine (C-P/O) or saline (S-P/O) injections were given 0.1 mg/kg pergolide, followed by 0.2 mg/kg ondansetron (s.c.) 3.5 h later for 5 consecutive days. The other half of the rats in the cocaine (C-D/S) and saline groups (S-D/S) received parallel vehicle injections for 5 consecutive days. All animals were then subjected to 9 days of withdrawal, followed by a challenge injection of 7.5 mg/kg cocaine (i.p.) (Fig. 1). Animals were then immediately returned to their home cages. The locomotion and behavioral rating scores were monitored over the next 60 min as previously described^28^. The baseline of locomotion in all animals was measured 1 day before cocaine injection. The behavioral score was given as 1 = asleep, 2 = almost asleep, 3 = dystonia, 4 = inactive, 5 = in-place oral behavior, 6 = grooming, 7 = normal-active movement, 8 = hyperactive, 9 = slow-patterned movement, 10 = fast-patterned movement, and 11 = stereotypy.

### Brain Dissection and Protein Measurement

Rats were euthanized and decapitated 24 h after the acute cocaine challenge. Brains were rapidly removed and sectioned for 1 mm coronal sections for medial prefrontal cortex (mPFC), caudate putamen (CPU), nucleus accumbens (NAc) core, NAc shell, and amygdala. Especially, mPFC was taken at +3.2 to +2.2 mm; CPU, NAc core, and NAc shell at +2.0 to +1.0 mm; and amygdala at −2.3 to −3.3 mm^28^. The tissue below the commissure was sectioned with the most ventral part designated as the NAc shell and the dorsal portion as the NAc core. Samples were stored at −80 ^o^C until protein extraction.

### BDNF Protein

BDNF levels in brain tissue lysates were measured using the BDNF Emax ImmunoAssay System kit (Promega, Madison, WI) according to the manufacturer’s instructions. The brain tissue lysates were diluted 1:4 with sample buffer provided with the kit. The BDNF standard curve was run for each plate (linear range: 7.8-500 pg/ml BDNF) and samples from a given brain area were determined in single assays.

### Western Blots

Western blots were performed as previously described^28^. The primary antibody [anti-ILK 1:1000; anti-phospho-Akt (Ser^473^)1:1000; anti-phospho-Akt (Thr^308^) 1:1000; anti-Akt 1:1000] and peroxidase-labeled secondary antibody (1:2000 dilution) were purchased from Cell Signaling Technology (Berverly, MA). The rabbit anti-TrkA, anti-TrkB antibodies and anti-p75^NTR^ antibody were purchased from Millipore (1:1000 dilution, Temecula, CA). The blot was developed with chemiluminescent substrate (Santa Cruz Biotechnology, Santa Cruz, CA). To control for loading efficiency, the blots were stripped and re-probed with α-tubulin antibody (1:1000 dilution; Abcam, Cambridge, MA) and expression levels of total ILK and phospho-Akt proteins were normalized to that of α-tubulin.

### ILK Activity Assay

ILK activity assay was conducted as previously described^29^, but with some modifications. Briefly, tissue lysates (100 μg) were incubated with 2 μg anti-ILK antibody (Millipore, Temecula, CA) or IgG as a negative control overnight at 4 °C, followed by addition of 50 μl protein A/G PLUS agarose beads (Santa Cruz Biotechnology) and continual incubation for 2 h at 4 °C. The immune complex was isolated by centrifugation, washed 5× with 1-ml homogenate buffer and then 2x subsequently with kinase reaction buffer. Kinase activity was determined by incubating the immunoprecipitated complex with 1 μg of inactive Akt-GST agarose (Millipore) and 200 μM of ATP in reaction buffer for 1 h at 30 °C. ILK activity was determined by Western blot of Akt-GST phosphorylation using the site-specific anti-phospho-Akt (Ser^473^) antibody (Cell Signaling).

### Immunofluorescence

After euthanization of rats and excision of rat brains, rat brains were soaked with 10% paraformaldehyde for 2 days and then with sucrose-infiltration solution (25% sucrose/PBS) for 24–30 hrs at 4 °C. The dual immunofluorescence staining was performed using free-floating coronal sections (15 μm). Briefly, sections were permeabilized with 10% normal goat serum containing 0.1% Triton X-100 for 2 hrs at room temperature. After rinsing sections briefly with PBS, sections were boiled in 10 mM citrate buffer (pH3.0) for antigen retrieval for 30 min and then incubated with primary antibodies [mouse anti-ILK antibody (1:100 dilution) plus rabbit anti-TrkA antibody (1:100), or rabbit anti-p75^NTR^ antibody (1:100)] overnight at 4 °C. After washing, sections were incubated with goat anti-rabbit Cy3 conjugated antibody (1:100, Millipore), or goat anti-mouse FITC conjugated antibody (1:100, Millipore) for 2 hr at room temperature, and then sections were washed with PBST and mounted with anti-fade medium (Vector Laboratories).

### Data analyses

The data were presented as means and standard errors of the mean and were analyzed using the Statistical Package for the Social Sciences, Version 20.0. The ambulation and biochemical results were analyzed using one-way ANOVA, while repeated measures ANOVA was used for the behavioral rating scores. The *post-hoc* analyses were performed using Bonferroni corrected pair-wise comparisons. A *p* < 0.05 was considered statistically significant.

## Results

### BDNF Changes in the mPFC and NAc Core Parallel Behavioral Sensitization to Cocaine

**Figure 2:**
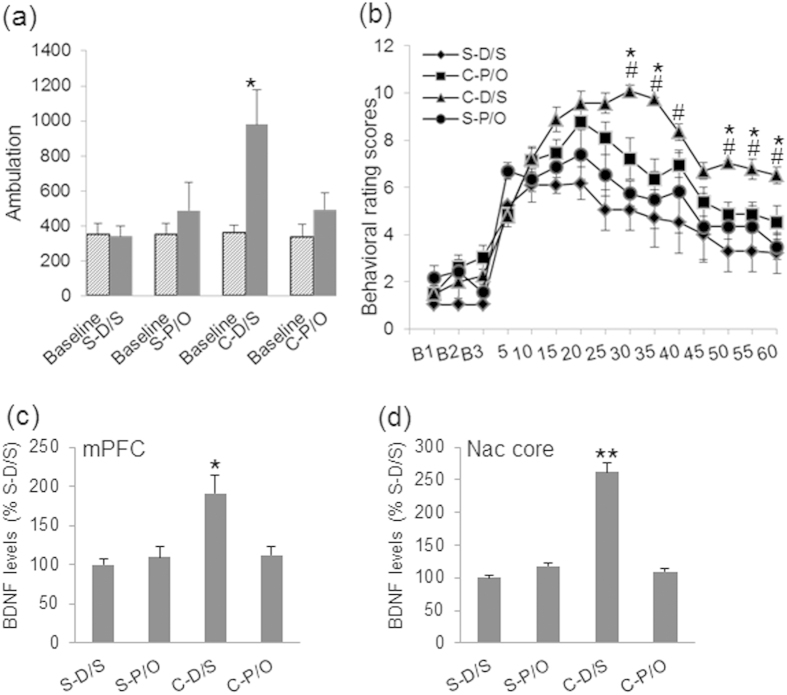
Pergolide/ondansetron (P/O) treatment blocked cocaine-induced behavioral sensitization and BDNF protein expression. Animals were treated as described in Materials and Methods. The cocaine sensitized rats (C-D/S) showed behavioral sensitization in ambulation (**a**) and behavioral rating scores (**b**) relative to the C-P/O and the saline controls (S-D/S and S-P/O). ^*^p < 0.01, C-D/S versus all groups; ^#^*p* < 0.05, C-D/S *vs* C-P/O. (**c**) BDNF levels in the mPFC were enhanced in the C-D/S group relative to all others. (*d*) BDNF levels in the NAc core were increased in the C-D/S group relative to the C-P/O and the two control (S-D/S and S-P/O) groups. See Figure 1 for details of the groups. *p < 0.05, **p < 0.01, C-D/S versus all groups. S-D/S, saline-DMSO/saline; S-P/O, saline-pergolide/ondansetron; C-D/S, cocaine-DMSO/saline; C-P/O, cocaine-pergolide/ondansetron.

ANOVA revealed significant effects of treatments on ambulation [F_(3,34)_ = 6.724, *p* < 0.001]. The C-D/S animals had significantly higher ambulation in response to cocaine challenge than the S-D/S (*p* < 0.001), S-P/O (*p* < 0.01), and C-P/O (*p* < 0.015) groups (Fig. 2a). The behavioral rating scores (Fig. 2b) were repeated measured by ANOVA. Significant main effects of time [F_(11,374)_ = 32.58, *p* < 0.001] and a significant time by treatment interaction [F_(33,374)_ = 3.86, *p* < 0.001) were observed. The Bonferroni test showed significantly higher scores in the C-D/S animals at 30, 35, 50, 55, and 60 min post-challenge compared to all other groups (*ps* < 0.05). Moreover, pergolide/ondansetron treatment (C-P/O) significantly reduced behavioral rating scores at 30, 35, 40, 50, 55, and 60 min compared to the C-D/S animals (*ps* < 0.05).

To determine whether these long-term changes in the brain could be blocked, BDNF levels in the mPFC, NAc core, NAc shell, CPU, and amygdala were measured using ELISA. No significant treatment effects were detected in the NAc shell, CPU, or amygdala (Table 1). In contrast, ANOVA revealed significant treatment effects on BDNF levels in the mPFC [*F*_(3,31)_ = 19.197, *p* < 0.001] and NAc core [*F*_(3,31)_ = 45.460, *p* < 0.001] (Fig. 2c,d). BDNF levels were significantly increased in the mPFC (p < 0.001) and NAc core (*p* < 0.001) in the sensitized (C-D/S) rats compared to rats in all other groups. Importantly, pergolide/ondansetron treatment (C-P/O) significantly normalized BDNF levels in the mPFC and NAc core. These findings suggest that: 1) cocaine sensitization selectively leads to long-term increases (>23 days after last cocaine injection, C-D/S group) in concentrations of BDNF in the mPFC and NAc core; and 2) combined pergolide/ondansetron treatment not only blocks the behavioral sensitization, but also normalizes BDNF levels in these two brain areas.

### Cocaine Sensitization Increases ILK Protein and Phospho-Ser^473^ Akt Levels in the mPFC and NAc Core

**Figure 3:**
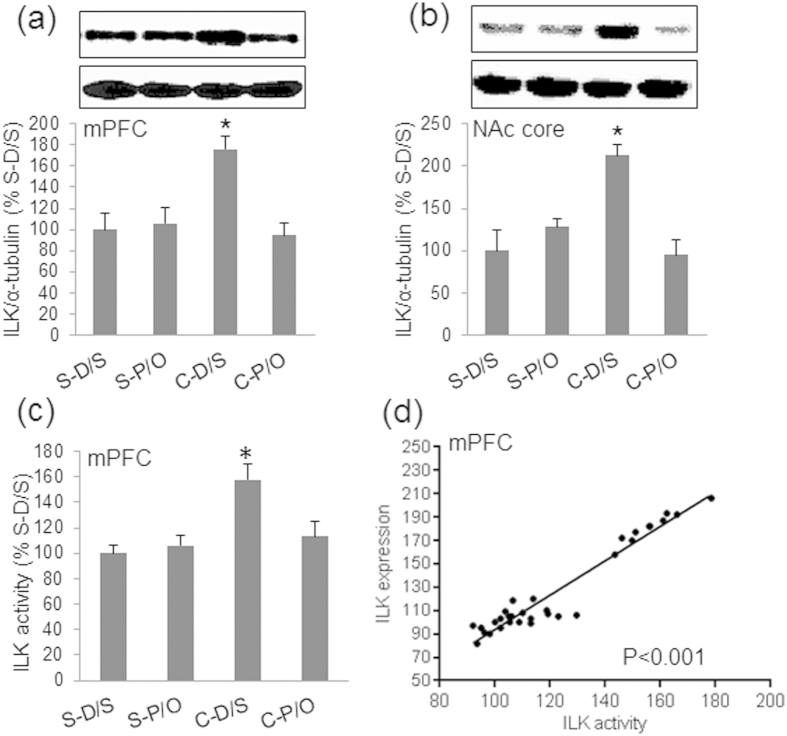
Pergolide/ondansetron treatment blocked changes in ILK expression following cocaine sensitization. (**a**) Percent densitometric values of integrin-linked kinase (ILK) protein levels in the mPFC in response to different treatment conditions; the top panel shows representative Western blots of ILK and α-tubulin proteins. Percent ILK protein levels are higher in the C-D/S groups than the other three groups. (**b**) Percent densitometric values of ILK levels in the NAc core; representative Western blots are shown in the top panel. Percent ILK protein contents are higher in the C-D/S group than in all other groups. See Figure 1 for details of the groups. *p < 0.05 C-D/S from all groups. (**c**) ILK activity and (*d*) correlation between ILK expression and ILK activity in the mPFC in saline-injected and cocaine-sensitized rats. Cocaine sensitization-induced increase in ILK activity correlated with increases in ILK expression. *p < 0.05, C-D/S versus all groups.

A previous study demonstrated that ILK is involved in cocaine sensitization^30^. Whether ILK levels parallel BDNF concentrations in the mPFC and NAc core has not been previously reported. Western blot showed that no changes were observed in ILK protein expression levels in the CPU, NAc shell, or amygdala, following establishment of cocaine sensitization (Table 1). In contrast, significant group differences were observed in the mPFC [*F*_(3,31)_ = 11.155, *p* < 0.001] and NAc core [*F*_(3,31)_ = 29.961, *p* < 0.001] (Fig. 3a,b). In the mPFC, ILK levels were significantly higher in the C-D/S group than in the S-D/S (*p* < 0.001), S-P/O (*p* < 0.001), and C-P/O groups (*p* < 0.001). Pergolide/ondansetron treatment (C-P/O) significantly normalized ILK levels in cocaine sensitized rats. Similar to the finding in the mPFC, ILK levels in the NAc core were significantly higher in C-D/S animals than in the other three groups (*p*s < 0.001) (Fig. 3b). We further investigated whether the changes in ILK expression resulted in parallel changes in ILK activity. Cocaine sensitization increased ILK activity (Fig. 3c, *p* < 0.05) which correlated with ILK expression (Fig. 3d) in the mPFC. This suggested that ILK activity is related to the ILK protein level.

**Figure 4:**
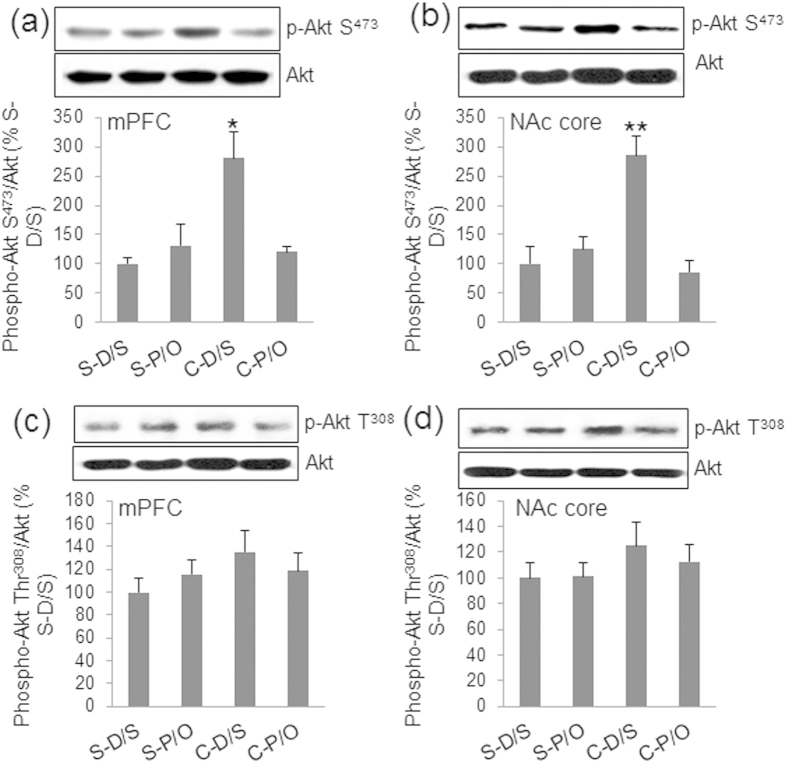
Alterations in the phosphorylation status of Akt in cocaine sensitized rats and its relationship with ILK. (**a**) Percent densitometric values of phospho-Ser^473^ Akt to total Akt in the mPFC are in the lower panel; the top panel shows representative Western blots of phospho-Ser^473^ Akt (p-Akt) and total Akt proteins. (**b**) Percent densitometric values of phospho-Ser^473^ Akt to total Akt in the NAc core in the bottom panel; representative Western blots are shown in the top panel. (**c**) Phosph-Thr^308^ Akt levels in the mPFC. (**d**) Phosph-Thr^308^ Akt levels in NAc core. See Figure 1 for details of the groups. Phospho-Akt protein levels are expressed as percent control where levels for the S-D/S group are set to 100%. *p < 0.05, **p < 0.01, C-D/S from all other groups.

Total Akt levels were not significantly altered among all animals in any tested brain areas (Table 1). In contrast, phospho-Ser^473^-Akt levels (normalized to total Akt protein) exhibited significant treatment effects in the mPFC and NAc core (Fig. 4a,b). One-way ANOVA revealed that levels of phospho-Ser^473^ Akt in the mPFC [*F*_(3,31)_ = 15.545, *p* < 0.001] and NAc core [*F*_(3,31)_ = 17.615, *p* < 0.001] were significantly different among treatment groups, but not in the CPU, NAc shell, or amygdala (Table 1). Bonferroni tests demonstrated that the relative levels of Akt Ser^473^ phosphorylation were increased in the mPFC of C-D/S rats compared to those in the S-D/S (*p* < 0.001) and C-P/O groups (*p* < 0.001), but not in the S-P/O group (Fig. 4a). In the NAc core, *post-hoc* analyses revealed that the level of Akt Ser^473^ phosphorylation was significantly higher in the C-D/S group than in all other groups (*p* < 0.001) (Fig. 4b). These changes in the levels of phospho-Ser^473^ Akt and ILK in the mPFC and NAc core parallel changes in the levels of BDNF protein in the same brain regions. In contrast, phosph-Thr^308^ Akt levels were not significantly different between groups in both the mPFC (Fig. 4c) and NAc core (Fig. 4d).

### Cocaine Sensitization Increases TrkB and p75^NTR^ Protein Levels in the mPFC and NAc Core

**Figure 5:**
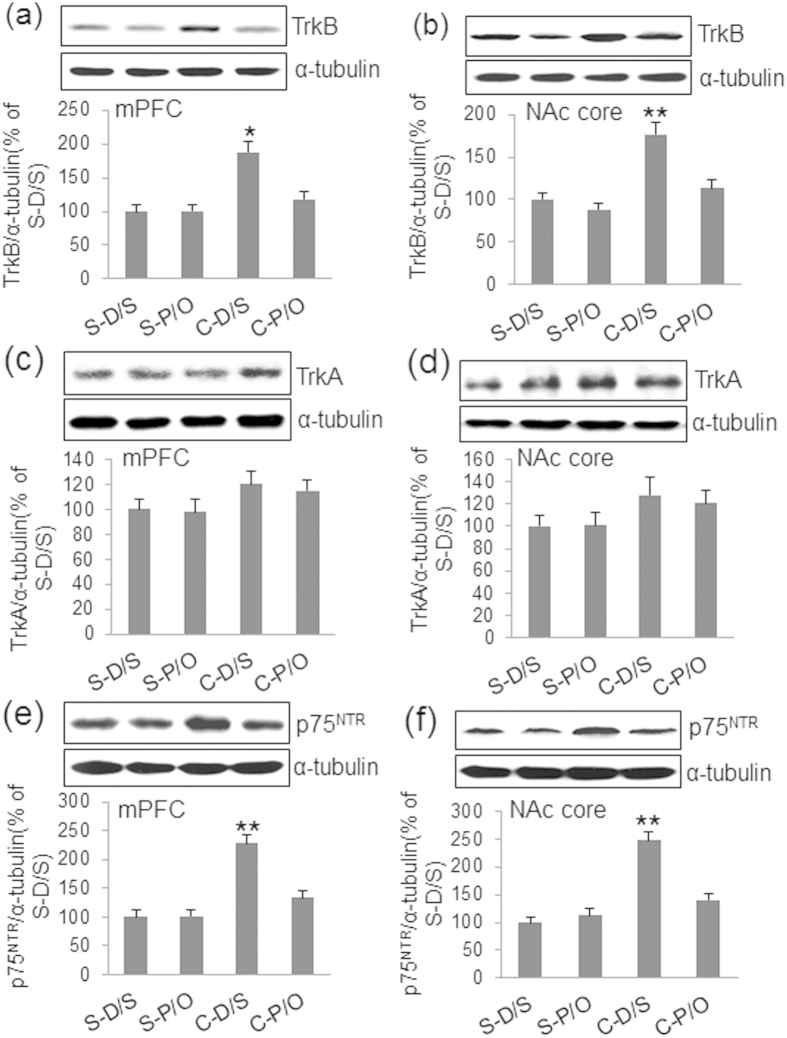
TrkA, TrkB, and p75^NTR^ expression in the mPFC and NAc core. (**a**) TrkB levels in the mPFC were enhanced in the C-D/S group relative to all others. (**b**) TrkB levels in the NAc core were increased in the C-D/S group relative to all other groups. TrkA levels in the mPFC (**c**) and NAc core (**d**) were not significantly different between groups. (**e**) p75^NTR^ level was increased in the mPFC in the C-D/S group relative to all others. (*f*) p75^NTR^ levels in the NAc core were increased in the C-D/S group relative to all other groups. See Figure 1 for details of the groups. *p < 0.05, **p < 0.01, C-D/S from all other groups.

It is well known that BDNF binds to the TrkB receptor and activates several signal pathways, including PI3K-Akt^11^. A previous study demonstrated that BDNF-mediated activation of ILK-Akt signaling requires co-expression of TrkA and p75^NTR^ receptors^12^. TrkA, TrkB, and p75^NTR^ expression were measured in brain tissues. No significant differences in TrkA, TrkB, and p75^NTR^ expression were observed in the CPU, NAc shell, or amygdala following establishment of cocaine sensitization (Table 1). In contrast, significant group differences in TrkB levels were observed in the mPFC [*F*_(3,31)_ = 11.155, *p* < 0.001] and NAc core [*F*_(3,31)_ = 19.961, *p* < 0.001] (Fig. 5A,B). In the mPFC, TrkB level was elevated in the C-D/S group relative to those in the S-D/S, S-P/O, and C-P/O groups (*p* < 0.001). The C-P/O group was not statistically different from either the S-D/S or S-P/O group (Fig. 5a). Similar to its levels in the mPFC, TrkB levels in the NAc core for C-D/S animals were enhanced compared to those in the other three groups (*p* < 0.001), which were not different from one another (Fig. 5b). In contrast, no significant group differences or significant differences in TrkA levels in the mPFC (Fig. 5c) and NAc core (Fig. 5d) between groups (p > 0.05) were observed. Significant group differences in p75^NTR^ levels were observed in the mPFC [*F*_(3,31)_ = 15.343, *p* < 0.001] and NAc core [*F*_(3,31)_ = 17.163, *p* < 0.001] (Fig. 5e,f). In the mPFC and NAc core, p75^NTR^ level was elevated in the C-D/S group relative to those in the S-D/S, S-P/O, and C-P/O groups (*p* < 0.001). The C-P/O group was not statistically different from either the S-D/S or S-P/O group (Fig. 5e,f).

### TrkA and p75^NTR^ co-locate with ILK Expression

**Figure 6:**
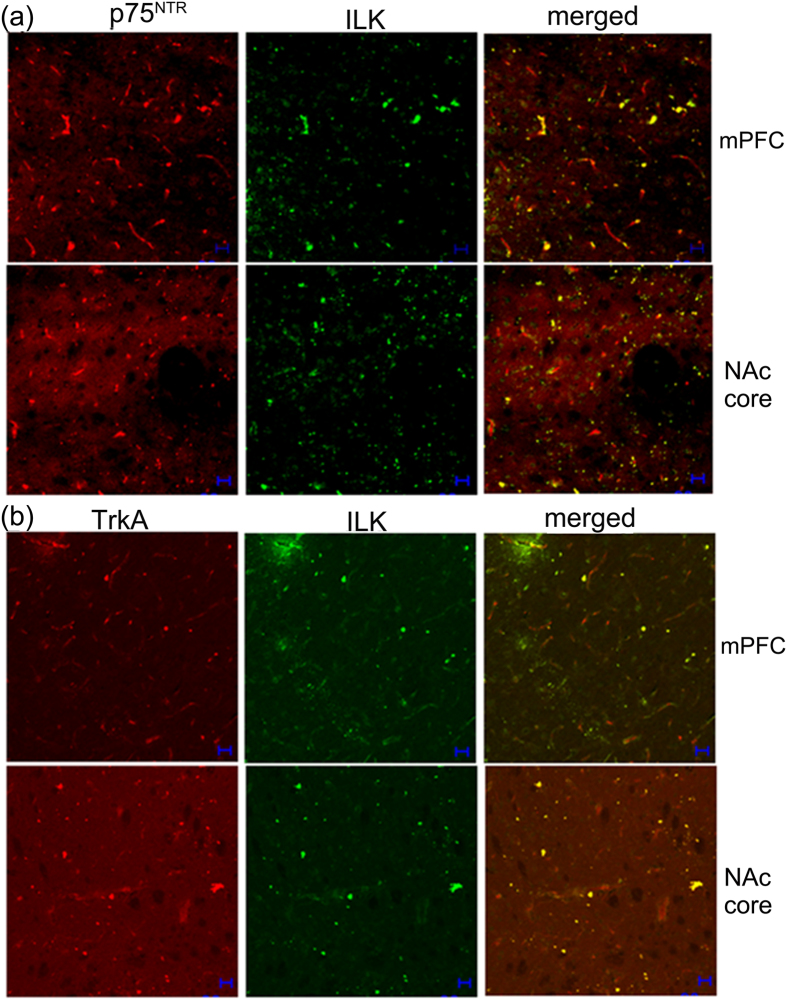
Colocalization of ILK with TrkA and p75^NTR^ expression. Coronal sections were dually immunofluorescence stained for ILK (green) and TrkA (red) or p75^NTR^ (red) expression. Fluorescent images were taken under Zeiss LSM 510 inverted confocal microscope. (**a**) ILK and p75^NTR^ expression in the mPFC and NAc core. ILK expression is colocalized with p75^NTR^ expression in both the mPFC and NAc core. (**b**) ILK and TrkA expression in the mPFC and NAc core. ILK expression is colocalized with TrkA expression in both the mPFC and NAc core. Scale bar = 20 μM.

Previous studies have demonstrated that ILK is localized to neuronal cell bodies and dendrites in various brain regions^21^. TrkA and p75^NTR^ are co-expressed on the cortical and striatal neurons^31^,^32^. A study in cell culture also demonstrated that BDNF can activate ILK-Akt through stimulating TrkA/p75^NTR^ heteroreceptor^12^. We therefore investigated whether the expression of TrkA and p75^NTR^ is co-located with ILK in the mPFC and NAc core, regions that showed changes in BDNF/ILK expression. The dual immunofluoresence staining revealed that TrkA and p75^NTR^ expression are co-distributed with ILK expression in the mPFC and NAc core (Fig. 6). This suggests that ILK might be activated by BDNF through stimulation of TrkA/p75^NTR^ heteroreceptor.

## Discussion

The present study has shown that long-term behavioral sensitization to cocaine is accompanied by increases in BDNF, TrkB, p75NTR, ILK, and phospho-Ser^473^ Akt protein levels in the mPFC and NAc core — two brain areas that have been demonstrated to be intimately involved in cocaine abuse. Moreover, combined pergolide/ondansetron treatment blocked either the behavioral alterations or the increases in the above signaling molecules. Although cocaine sensitization is known to produce long-lasting changes in behavior and molecules related to synaptic plasticity^19^,^33^,^34^, our results first suggested an involvement of the BDNF-TrkA/p75^NTR^-ILK-Akt signaling pathway in cocaine-induced long-lasting behavioral alterations. Importantly, both the sensitization-associated behaviors and molecules could be blocked by the clinically available drugs.

A previous study in PC-12 cells demonstrated that BDNF-mediated activation of ILK-Akt signaling requires co-expression of TrkA and p75^NTR^ receptors^12^. *In vivo* studies have shown the co-expression of TrkA and p75^NTR^ receptors on the cortical and striatal neurons^31^,^32^. TrkA and p75^NTR^ receptors were thought to often exist on the same neurons, coordinating and modulating neuronal neurotrophin responses by suppressing or enhancing each other’s actions^32^,^35^ or by reciprocally modulating the receptor affinity states in the TrkA and p75^NTR^ heteroreceptor complex^36^. However, whether the cocaine sensitization is also associated with the activation of BDNF-ILK-Akt signaling, particularly, through the activation of TrkA and p75^NTR^ heteroreceptor remains inadequately addressed. Consistent with previous studies stated above, this study demonstrated that p75^NTR^ and TrkA receptors were co-located in the NAc core and mPFC, and were also co-located with ILK expression in these two brain areas. However, Western blot demonstrated that p75^NTR^, but not TrkA expression was upregulated in the mPFC and NAc core, paralleling the increases in ILK expression and Akt Ser^473^ phosphorylation. Therefore, we hypothesized that: 1) BDNF-dependent activation of ILK-Akt signaling in the mPFC and NAc core may be mediated through TrkA/p75^NTR^ heteroreceptors. It requires only the coordinate increase in p75^NTR^ activation. In contrast, TrkA may only be required to be co-expressed as a coordinator; and 2) enhanced p75^NTR^ activation and subsequent ILK-Akt signaling may play a significant role in cocaine behavioral sensitization and its reversal by combined pergolide/ondansetron treatment.

It is well established that BDNF specifically binds to the tyrosine kinase B (TrkB) receptor and activates various signaling cascades, which include PI3K-dependent activation of Akt where the Thr^308^ and Ser^473^ residues become phosphorylated^7^,^37^. In this study, consistent with increases in BDNF concentrations, cocaine sensitization parallels increases in the levels of TrkB and phospho-Ser^473^ Akt, but not phospho-Thr^308^ in the mPFC and NAc core. Among signaling molecules that regulate Akt phosphorylation, ILK is one of the upstream kinases that can phosphorylate Akt at the Ser^473^ residue^38^. The present study demonstrated that changes in ILK protein levels parallel those in the levels of BDNF and phospho-Ser^473^ Akt following establishment of sensitization. In addition, the change in ILK protein levels parallel the change in ILK enzymatic activity *in vivo*. A previous study demonstrated that established cocaine sensitization is associated with opposite changes in the PI3K activity levels in the mPFC and NAc core, but pergolide/ondansetron treatment fails to block either of these changes^19^. These divergent alterations in the status of PI3K are quite different from the parallel changes observed in BDNF-ILK-Akt Ser^473^ signaling and behavioral sensitization in the present study. Taken together, these observations suggest that: 1) BDNF is an initiator of ILK-Akt signaling which may not need the corresponding changes in TrkB-PI3K signaling to become activated. In contrast, it may only require constitutive PI3K activity to become activated although pergolide/ondansetron treatment blocked TrkB levels in rats that were administered cocaine; and 2) dysregulation of BDNF-TrkA/p75^NTR^-ILK-Akt signaling may play a significant role in long-term maintenance of cocaine sensitization.

While the discrepancy between PI3K- and ILK-mediated signaling events may be attributed to a number of different factors, the present study suggests that these two events are mediated by different neurotrophin receptors and thus are independent of each other although constitutive PI3K activity may be required. Other PI3K-independent pathways could also participate in BDNF-Akt signaling during cocaine sensitization and its reversal. These transduction pathways may include MAPK; however, in this case, Akt activation is primarily achieved through Thr^308^ phosphorylation^39^. Finally, the increased Akt signaling may also be due to an as yet-to-be-identified non-BDNF-dependent signaling molecule. For instance, NMDA or AMPA receptor stimulation also leads to phosphorylation of Akt^40^,^41^. Long-term cocaine sensitization is associated with brain region-dependent alterations in phosphorylation of the NR2B and GluR1 subunits of the respective NMDA and AMPA receptors, and these changes are blocked by pergolide/ondansetron treatment^28^. However, this study provided no further evidence on how BDNF was activated and how the BENF-TrkA/p75^NTR^-ILK-Akt signaling was involved in the long-term behavioral sensitization to cocaine.

Previous studies have demonstrated that addictive drugs induce cAMP increase via activation of dopamine receptors and calcium entry via activation of glutamate NMDA receptors. These events, in turn, activate numerous intracellular signaling cascades^7^,^42^,^43^. Among them, activated CREB contributes to regulation of genes important to the neuroplastic changes, including regulation of BDNF mRNA transcription, translation, and translocation to dendrites^9^,^44^,^45^,^46^. BDNF is secreted locally near active synapses and binds to Trk receptors located on presynaptic and postsynaptic sites. The activated BDNF/Trks signaling can also phosphorylate AMPA receptors and subsequently activates the CREB and may result in progressive BDNF gene expression and release^44^,^47^. There are many ways to interfere with signaling involved in BDNF transcription and translation, which subsequently affects BDNF levels and the sensitization-associated synaptic plasticity. Here, pergolide is a DA agonist and its behavioral effects can last for 3-4 hrs in animals^48^. Previous studies showed that pergolide alone was not sufficient to consistently block the sensitization-associated behavioral and molecular changes^28^,^49^. However, pergolide can induce an ‘acute DA withdrawal state’^28^. 5-HT_3_ terminal receptors were thought to mediate local stimulatory actions of 5-HT on DA release in the mPFC, striatum, and NAc^28^. Ondansetron is a 5-HT_3_ antagonist. A previous study suggested that pergolide may evoke a methamphetamine associated memory and that ondansetron can disrupt its reconsolidation^50^. Thus, modulations of the DA–5-HT_3_ interactions in the mPFC and NAc core may form a basis for blocking the sensitization-associated behaviors and BDNF/ILK/Akt changes by a pergolide/ondansetron treatment regimen.

In addition to changes in signal transduction, chronic abuse of drugs (e.g., cocaine) can produce persistent structural alterations in the cytoskeleton. These changes include increases in dendritic branching and density of dendritic spine on medium spiny neurons in the NAc and pyramidal cells in the PFC^51^,^52^,^53^. BDNF stimulation can also lead to similar structural changes^54^,^55^. Furthermore, ILK can regulate integrin-dependent neurite outgrowth in N1E-115 neuroblastoma cells^23^ and NGF-stimulated differentiation of PC-12 cells and dorsal root ganglion neurons^21^. These observations suggest that increased BDNF levels may be associated with the morphological plasticity observed in long-term psychostimulant (cocaine) sensitization through the BDNF-ILK signaling cascade. Precise relationships of this neurobiological process with functional and morphological changes in behavioral sensitization await further elucidation.

In conclusion, this study suggests that the BDNF-TrkA/p75^NTR^-ILK-Akt signaling pathway may be active in cocaine sensitization and could be associated with neural plasticity in the mPFC and NAc core.

## Additional Information

**How to cite this article**: Zhang, Y. *et al*. Molecular changes in the medial prefrontal cortex and nucleus accumbens are associated with blocking the behavioral sensitization to cocaine. *Sci. Rep*. **5**, 16172; doi: 10.1038/srep16172 (2015).

## Figures and Tables

**Table 1:** Negative outcomes in molecular changes.

Molecules*	Brain areas	S-D/S	S-P/O	C-D/S	C-P/O
BDNF levels	CPU	100.0 ± 9.7	115.5 ± 16.5	118.4 ± 18.6	113.9 ± 13.4
	NAc shell	100.0 ± 9.4	106.5 ± 13.7	108.4 ± 12.4	106.8 ± 11.7
	Amygdala	100.0 ± 10.7	99.5 ± 16.5	111.5 ± 10.6	109.6 ± 11.2
ILK Protein	CPU	100.0 ± 9.0	105.9 ± 15.7	111.3 ± 16.6	98.8 ± 14.3
	NAc shell	100.0 ± 9.6	105.8 ± 10.5	108.9 ± 14.1	100.8 ± 11.2
	Amygdala	100.0 ± 9.7	100.9 ± 11.4	111.1 ± 12.2	98.9 ± 10.1
Total Akt	CPU	100.0 ± 11.3	113.6 ± 17.6	121.3 ± 18.9	117.9 ± 18.9
	NAc shell	100.0 ± 9.9	108.4 ± 13.5	109.6 ± 17.1	102.7 ± 15.4
	Amygdala	100.0 ± 10.4	97.4 ± 11.4	104.9 ± 12.4	99.8 ± 10.1
	mPFC	100.0 ± 10.8	118.5 ± 14.4	120.9 ± 18.1	107.8 ± 16.6
	NAc core	100.0 ± 11.7	107.7 ± 15.4	114.9 ± 18.2	110.5 ± 17.3
p-Akt-ser^473^	CPU	100.0 ± 12.1	115.4 ± 16.3	120.1 ± 19.4	118.5 ± 17.6
	NAc shell	100.0 ± 11.4	116.4 ± 14.7	118.9 ± 15.9	109.4 ± 13.2
	Amygdala	100.0 ± 12.9	109.2 ± 13.4	118.8 ± 18.5	99.9 ± 16.6
p-Akt-Thr^308^	CPU	100.0 ± 11.1	105.8 ± 15.4	110.3 ± 17.5	100.7 ± 16.5
	NAc shell	100.0 ± 12.1	110.0 ± 13.7	110.7 ± 14.3	104.3 ± 13.8
	Amygdala	100.0 ± 10.9	99.7 ± 12.9	106.9 ± 15.3	100.9 ± 13.4
TrkA	CPU	100.0 ± 10.9	100.9 ± 14.6	109.5 ± 15.7	100.3 ± 15.2
	NAc shell	100.0 ± 9.9	99.0 ± 11.3	103.6 ± 12.6	101.8 ± 11.2
	Amygdala	100.0 ± 10.4	98.7 ± 12.1	109.0 ± 13.1	101.1 ± 13.0
TrkB	CPU	100.0 ± 12.3	106.4 ± 13.6	108.8 ± 16.0	103.5 ± 14.7
	NAc shell	100.0 ± 9.9	100.5 ± 10.9	105.8 ± 12.5	102.5 ± 11.3
	Amygdala	100.0 ± 10.4	106.9 ± 14.6	112.2 ± 15.3	110.7 ± 14.3
p75^NTR^	CPU	100.0 ± 12.4	100.4 ± 16.4	110.0 ± 17.4	104.4 ± 13.8
	NAc shell	100.0 ± 12.3	102.6 ± 14.5	108.5 ± 15.5	108.2 ± 16.0
	Amygdala	100.0 ± 11.9	109.2 ± 15.7	114.3 ± 18.1	115.1 ± 15.8

^*^ANOVA showed no significant group differences (p > 0.05). CPU: caudate putamen; NAc: nucleus accumbens; mPFC: medial prefrontal cortex.
